# Genetic Diversity, Virulence, and Antibiotic Resistance Determinants of *Campylobacter jejuni* Isolates in Romania

**DOI:** 10.3390/pathogens13090716

**Published:** 2024-08-23

**Authors:** Madalina Baltoiu, Gratiela Gradisteanu Pircalabioru, Daniela Cristea, Marilena Sorokin, Cristiana Cerasella Dragomirescu, Ileana Stoica

**Affiliations:** 1 Cantacuzino National Military Medical Institute for Research and Development, Independence Spl. no. 103, 050096 Bucharest, Romania; militaru.madalina@cantacuzino.ro (M.B.); ceraseladragomirescu@yahoo.com (C.C.D.); 2 Faculty of Biology, University of Bucharest, Independence Spl. no. 91-95, 050095 Bucharest, Romania; Ileana.stoica@bio.unibuc.ro; 3 eBio-Hub Research Centre, National University of Science and Technology Politehnica Bucharest, Iuliu Maniu Boulevard, 061344 Bucharest, Romania; 4 Department of Public Health Prahova, Maramures Street, 100029 Ploiesti, Romania; marilena_sorokin@yahoo.com; 5 Department of Microbiology, Cantacuzino Institute, Carol Davila University of Medicine and Pharmacy, 020021 Bucharest, Romania

**Keywords:** *Campylobacter*, *C. jejuni*, virulence, antibiotic resistance

## Abstract

The emergence of antibiotic-resistant *Campylobacter jejuni*, a leading cause of gastroenteritis worldwide, presents a significant public health challenge requiring vigilant surveillance and disease control. This study aimed to characterize *C. jejuni* strains isolated in Romania from 2017 to 2020, focusing on genetic diversity, virulence, and antibiotic resistance determinants. The isolates underwent phenotypical testing, PCR, and antibiotic resistance assessment using the Kirby–Bauer disc diffusion method for ciprofloxacin, erythromycin, and tetracycline. Genetic analysis identified resistance and virulence genes, point mutations, and performed sequence typing (7-gene MLST) to determine genetic relatedness. Results indicated substitutions at position 86 in the amino acid sequence or position 257 in the nucleotide sequence of the *gyrA* gene in 47 fluoroquinolone-resistant isolates. Additionally, mutations in the *rRNA 23S* gene at positions 2074 and 2075, associated with macrolide resistance, were found in 12 of the 66 isolates. Allelic profiles generated 38 sequence types (STs), including three new STs not present in the reference database. The sequence data analysis revealed a genetically diverse *C. jejuni* population with a weak clonal structure. This study provides crucial insights into the genetic diversity and antibiotic resistance of *C. jejuni* strains in Romania, highlighting the need for ongoing surveillance and control measures.

## 1. Introduction

Campylobacteriosis was the most reported zoonosis and foodborne illness of 2022 in EU. A total of 140,241 cases were reported, compared to 129,960 the previous year, with a notification rate of 46.9. The countries with the highest rates are Czechia and Luxembourg while the lowest rates were reported in Bulgaria, Greece, Poland, and Romania. The case fatality rate is low, accounting for 0.04% of the reported cases, compared to Listeriosis and West Nile virus infection with a percentage of 18.1% and 8.3%, respectively [1,2].

One of the main causative agents of gastroenteritis worldwide is *Campylobacter jejuni*, which is transmitted to humans through the consumption of raw or undercooked meat, milk, and dairy products or cross contamination events [3,4,5]. Another important risk factor is international travel, especially in the Southeast parts of Asia [3]. Moreover, pets and contaminated water were also reported as being sources of infection [6]. Although the illness lasts for only a few days and is an acute and self-limiting infection, it may cause severe and prolonged symptoms in young and elderly patients. This can lead to complications such as the post-infection immune disorder Guillain–Barre syndrome, Miller–Fisher syndrome, meningitis, brain abscess, sepsis, endocarditis, myocarditis, reactive arthritis, inflammatory bowel disease, celiac disease, cholecystitis, and colon cancer [7], thus requiring antibiotic treatment [8]. The most commonly used antibiotics are macrolides and fluoroquinolones. Tetracycline and gentamycin are used as alternative treatments when the conventional treatment is not efficient [7].

Multidrug resistant *C. jejuni* exhibits resistance against erythromycin, fluoroquinolones, and tetracycline. The use of antibiotics as prophylaxis in livestock seems to be the main cause of antibiotic resistance acquisition, either through horizontal gene transfer from other bacteria or mutations [6].

A single-point mutation within the quinolone resistance region–QRDR–*gyrA* is sufficient to confer resistance to fluroquionolones. One mutation frequently associated with AR is C-257 to T (Thr86Ala). Other single-point mutations, such as Tr86Ala, Ala70Thr, and Asp90Asn were described [9]. Furthermore, mutations in the *gyrA* gene are not only responsible for the resistance to fluroquinolones, but they also increase the virulence of strains [9]. Another mechanism involved in the decreased susceptibility to these antibiotics is the activity of the CmeABC efflux pump [7].

Most commonly, resistance to macrolides is acquired due to a chromosomal mutation in the V domain of the *23S rRNA gene*. Other mechanisms of resistance to macrolide include the presence of the gene *ermB* (rRNA methyltransferase gene) and point mutations in L4/L22 encoding ribosomal proteins [7].

To date, more than 60 tetracycline resistance genes have been described, most of them located on plasmids [10]. Mosaic *tet* genes are common, a vast majority of them being derived from *tet(O)*, *tet(W)*, *tet(S)*, *tet(M)*, and *tet32*. The resistance levels of these genes are often similar to the non-mosaic genes; however, *tet(O/W/32/o/w/o)* confers a higher level of resistance than the original genes [10]. There are similarities between *tet(O/32/O)* and *tet(O/M/O)*, namely the length and insertion position of *tet32* and *tet(M)* within *tet(O)*, thus distinguishing between them is rather difficult [10].

The aim of this study was to propose a molecular analysis of *Campylobacter jejuni* isolates from patients in Romania, which associates the description of the essential aspects for a human pathogen—its intrinsic virulence and the development of mechanisms of resistance to the bactericidal action of antibiotics—with intraspecific discrimination based on ST type (sequence type) by molecular typing MLST (Multi-Locus Sequence Typing).

## 2. Materials and Methods

### 2.1. Bacterial Isolates

This study was performed in a National Center for Enterobacteria—Laboratory for bacterial enteric infections. A total of 66 *C. jejuni* strains isolated between 2017 and 2020 from patients with acute diarrheal disease were included in the study. The isolates were provided by the Department of Public Health located in two counties from Romania. *C. jejuni* strains were recovered on Columbia enriched blood agar media (7%) and incubated for 48 h at 42 °C in a micro aerobic atmosphere (85% N_2_, 10% CO_2_, and 5% O_2_) using Campygen Compact sachets (Thermo Fisher Scientific Oxoid Ltd., Basingstoke, UK). The species were confirmed using Matrix-Assisted Desorption/Ionization-Time of Flight Mass Spectrometry (MALDI-TOF MS) Bruker MALDI Biotyper (Bruker Daltonik GmbH Biotyper Identification, Bruker BioSciences Corporation, Bremen, Germany).

### 2.2. Antimicrobial Susceptibility

The antimicrobial susceptibility testing to tetracycline (TE–30 μg), ciprofloxacin (CIP–5 μg), and erythromycin (E–15 μg) was performed by using the Kirby–Bauer disc diffusion method according to the protocol of the European Committee on Antimicrobial Susceptibility Testing (EUCAST) for fastidious organisms, using the ATCC 33560 *C. jejuni* as a quality control.

### 2.3. Genetic Support of Resistance and Virulence

The strains were investigated for genes and point mutations conferring antibiotic resistance. Template DNA was prepared using the Invitrogen PureLink Genomic DNA mini kit (Thermo Ficher Scientific, Carlsbad, CA, USA) according to the manufacturer’s instructions and quantified using the Qubit™ 3.0 fluorometer (Thermo Fisher Scientific, Carlsbad, CA, USA) and Qubit^®^ dsDNA HS Assay Kit (Invitrogen, Waltham, MA, USA). The primers (DNA Technology Biozyme, Biozyme, Cluj Napoca, Romania) used for amplifying virulence and resistance genes and their annealing temperatures are presented in Table 1 [11,12,13,14,15,16,17]. The amplification reactions were carried out in a final volume of 50 µL, using 2 µL of DNA and 1.5 U Taq polymerase (Promega, Madison, WI, USA) and 2x PCR master mix (Promega, Madison, WI, USA).

The presence of tetracycline resistance gene *tetO* was detected using a PCR-based protocol with primers described by Gibreel et al. [18]. Also, screening of mutations conferring resistance to macrolides and fluoroquinolones, respectively, was performed. The primers used for amplification and sequencing of two common mutations (i.e., A2074C and A2075G) in the *23S rRNA* gene conferring macrolide resistance were as described by Alonso et al. [17]. The QRDR of the *gyrA* gene was amplified and sequenced using the primers previously described by Zirnstein et al. [16].

PCR assays for genes associated with virulence were performed using previously published specific primers targeting *cadF*, *ceuE*, *virB11*, *flaA*, and *cdt* genes (A, B, C).

The amplicons were purified with the NucleoSpin Gen kit and PCR Clean-up-Macherey Nagel, as recommended by the producers and quantified with Qubit Fluorometer 3.0 (Thermo Fisher Scientific, Carlsbad, CA, USA). DNA sequences were determined by primer pairs gyrA7/gyrA8 for the mutation in the *gyrA* gene associated with fluoroquinolone resistance and primers 23SRNA-F/23SRNA-R for the mutation at position 2074 and 2075 associated with erythromycin resistance, using BigDye Terminator v3.1 (Applied Biosystems, Thermo Fisher Scientific, Carlsbad, CA, USA), according to the manufacturer’s instructions and purified using the DyeEx 2.0 Spin kit colonies (Qiagen, Hilden, Germany). Separation and detection of sequences were performed using a SeqStudio Genetic Analyzer (Applied Biosystems, Thermo Fisher Scientific, Carlsbad, CA, USA), the obtained sequences were edited and analyzed with the BioEdit Sequence Alignment Editor program, version 7.0.5.3 (free software, Tom Hall).

*C. jejuni* strains both sensitive and resistant to fluoroquinolones were compared and analyzed. The detection of mutations in the amino acid sequence at position 86 in the *gyrA* gene was compared with the sequence complete *C. jejuni* UA580 *gyrA* in the database with the accession number L04566 in GenBank [19], and also erythromycin resistance confirmed the presence of a point mutation in position 2074 (A–C) and 2075(A–G) using wild type reference sequence of *C. jejuni* NCTC 11168 23S ribosomal database [17].

### 2.4. Multilocus Sequence Typing (MLST)

Nucleotide sequence analysis of internal fragments of seven housekeeping genes (i.e., *aspA*, *glnA*, *gltA*, *glyA*, *pgm*, *tkt*, and *uncA*) was performed as described by Dingle et al. [20]. Primers used for amplification and sequencing reactions are shown in Table 2, providing reliable amplification for *C. jejuni* isolates. Sequencing of the amplified PCR products was done on a SeqStudio Genetic Analyzer (Applied Biosystems, Thermo Fisher Scientific, Carlsbad, CA, USA) in both orientations. Allele numbers for each housekeeping gene, sequence types (STs), and clonal complexes (CCs) were assigned by submitting the DNA sequences to the *C. jejuni* MLST database.

The mixture for the amplification reactions was made in a final volume of 50 µL with 10 ng DNA, 1 µL of each primer at a concentration of 10 µM (DNA Technology Biozyme), 5 µL 1× reaction buffer,1 µL dNTP mix 10 mM, 1.5 mM MgCl2, and 1.5 U Taq DNA polymerase/reaction (Promega). Amplification conditions were as follows: 95 °C for 3 min, followed by 35 cycles of 94 °C for 2 min, 50 °C for 1 min, and 72 °C for 1 min, with a final elongation at 72 °C for 5 min. For each locus, the sequences of the distinct alleles were assigned arbitrary allele numbers in order of identification, these being internal fragments (proximately 400–500 bp) within a gene containing an exact number of codons. Each isolate was designated with seven distinct numbers, constituting an allelic profile or sequence type [20].

## 3. Results

The analysis of *Campylobacter jejuni* strains isolated from Romania between 2017 and 2020 yielded significant insights into the genetic diversity, virulence, and antibiotic resistance profiles of these pathogens. A combination of phenotypical testing, PCR, and antibiotic susceptibility testing provided a comprehensive overview of the resistance mechanisms and genetic relatedness of the isolates.

### 3.1. Virulence Genes

The results obtained for the seven genes encoding virulence factors and toxins specific to *Campylobacter jejuni* strains isolated from fecal samples (sporadic cases of gastroenteritis complicated with diarrhea from children aged between 3 months and 9 years provided by the Public Health in our country), were analyzed and compared and are presented in Table 3.

A single specific amplicon for the *flaA* gene corresponding to the expected size of 1728 bp was generated for all 66 *C. jejuni* strains (100 %); similar results were also obtained for the *cadF* (100 %) and *ceuE* genes, respectively (100 %). In the case of the the *virB11* gene, 14 isolates (21 %) from the 66 strains *of C. jejuni* were positive.

Primers used for the detection of cdt (cytolethal distending toxin) genes *cdtA*, *cdtB*, and *cdtC* were obtained in all *C. jejuni* isolates analyzed. The *cdtA* gene PCR product with an expected size of 165 bp was observed in 62 *C. jejuni* isolates (93%), *cdtB* gene sequences were detected in 98% of them, and *cdtC* gene amplicons with an expected size of 555 bp were also observed in 98% of them.

### 3.2. Resistance

In this study, 47 isolates of *C. jejuni* with increased resistance to fluoroquinolones with an inhibition diameter < 26 mm were selected and analyzed. After sequencing, the data obtained determined the substitution in position 86 in the amino acid sequence Supplementary Figure S1, or in position 257 in the nucleotide sequence of *gyrA* gene due to a DNA codon mutation from ACA (Thr-threonine) to ATA (Ile-isoleucine) Supplementary Figure S2.

Out of 12 strains (18%) that were phenotypically resistant to erythromycin, data obtained by sequencing confirmed the presence of a point mutation in position 2075 (A–G) in all 12 isolates of *C. jejuni*, with 5 isolates harboring a double mutation (2074 A–C, 2075 A–G), while in the other 5, only the 2074 single point mutation was detected Supplementary Figure S3.

Results of tetracycline susceptibility testing determined that 55% of clinical isolates (36 out of 66 samples) showed resistance to tetracycline, whereas 45% of the isolates showed sensitivity to this class of antibiotics. Therimers DMT1 and DMT2 (Invitrogen) produced a 559 bp amplicon for 36 *C. jejuni* strains specifically for the *tetO* gene responsible for tetracycline resistance. The phenotypically sensitive strains did not generate amplification for this gene.

In this study, virulence and resistance profiles to antibiotics such as fluoroquinolones, macrolides, and tetracyclines, classes of antibiotics that are often used first-line in the treatment of infections with *C. jejuni* strains, were observed.

### 3.3. Sequence Typing

For each locus, distinct allele sequences were assigned, these being internal fragments of the gene containing an exact number of codons; each isolate was designated with seven numbers constituting an allelic profile or ST (sequence type).

ST types were identified by numbers assigned to alleles and compared in the database; these were grouped into clonal complexes [21] Supplementary Figure S4.

The length and frequency of the alleles defined by the MLST scheme are shown in Table 4, namely *aspA-477* bp, *glnA-477* bp, *gltA-402* bp, *glyA-507* bp, *pgm-498* bp, *tkt 459* bp, and *unc-489* bp. From the 66 *C. jejuni* isolates, 10 strains were included in CC (clonal complex) 257 (9 strains ST824, 1 strain ST2254), 10 strains were included in CC353 (4 strains ST400, 4 strains ST353, and 2 strains in ST356), 5 strains in CC21 were included in ST50, 4 strains were included in CC 443 (ST51), and the rest of the strains were included in other types of clonal complexes.

**Figure 1:**
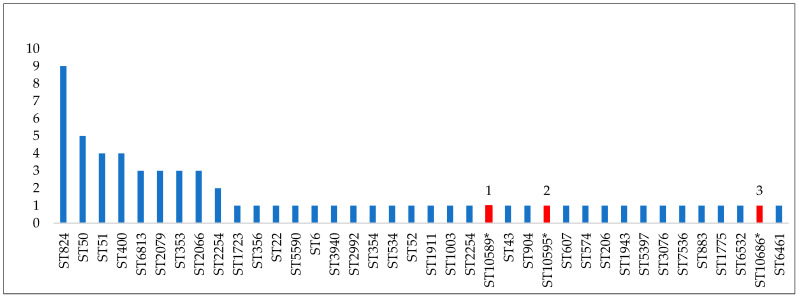
The 38 types of sequences identified by the MLST technique (1, 2, and 3 new ST, shown in red).

The allelic profiles obtained generated 38 sequence types existing in the database of which 3 were new STs of *Campylobacter jejuni* (10589, 10595, 10686) Supplementary Table S1, absent in the database used to establish the ST type and entered in the database international (pubmlst.org) having accession numbers 106266, 106267 and 106791 (Figure 1).

The most common type sequences found among *C. jejuni* isolates in this study were ST824 (9 strains), ST50 (5 strains), ST51 (4 strains), ST400/ST353 (4 strains), ST2079, and ST2066 (3 strains) (Table 5)

The strains of *C. jejuni* in this study were typed by this method. The allelic profiles obtained generated various types of ST (sequence type) already known. These data indicated a genetically diverse population, having a weak clonal structure.

## 4. Discussion

In this study, we analyzed the sequence data of antibiotic resistance and virulence determinants collected in Romania. Furthermore, the relatedness of the strains was assessed.

*Campylobacter* infection is generally considered a self-limiting disease that does not require therapeutic intervention, but in the case of severe complications, antibiotics are recommended, especially in patients with immunodeficiency [22]. Drugs used in the clinical therapy of campylobacteriosis are macrolides (such as azithromycin and erythromycin, especially in children) and fluoroquinolones (ciprofloxacin—occasionally used in pediatrics). Tetracyclines are considered an alternative choice in the therapy of Campylobacter infections, but in practice they are not often used [23,24]. The use of antimicrobial agents as food additives in animals to control their infections has contributed to an increased resistance of microorganisms against antibiotics. The uncontrolled administration of fluoroquinolones to poultry has contributed to the increase in the resistance of *C. jejuni* to fluoroquinolones in industrialized areas [25].

To our knowledge, data available on the genetic analysis of antibiotic-resistant *C. jejuni* strains recovered from human campylobacteriosis cases in Romania is very scarce. Annually, up to 10% of the population is affected by foodborne diseases according to WHO.

The prevalence for the seven virulence genes and toxins of the analyzed *C. jejuni* strains was determined by PCR. The sequences for the three virulence genes (*cadF*, *flaA*, *ceuE*) as well as the *cdtC* toxin were detected in 98–100% of the isolates analyzed in this study.

A high prevalence of 100% of the *cadF*, *ceuE*, and *flaA* gene was consistent with similar results of previous studies using identical PCR detection assays [11]. This high prevalence may be explained by the fact that the *cadF* gene represents an important virulence factor as well as the *flaA* gene that plays an important role in *Campylobacter* pathogenesis because *Campylobacter* motility requires flagellum production.

The low prevalence of 21% of the *virB11* gene among the isolates analyzed compares with studies reported by Bacon et al., 2000 [13], the low virulence being due to isogenic mutations. VirB-D—plasmid-mediated—is associated with plasmid pVir [13,26]. Sequences obtained were aligned and compared with the sequence from the database (*C. jejuni* UA580 gyrA accession number L04566) and determined the substitution in position 86 in the amino acid sequence/position 257 in the nucleotide sequence of the *gyrA* gene for 47 *C. jejuni* isolates. This substitution is due to a DNA codon mutation from ACA (Thr-threonine) to ATA (Ile-isoleucine). Our results are similar to those of Pham et al., 2016 [27].

Also tested were three sensitive strains having an inhibition diameter ≥50 mm around antibiotic discs, and these did not show the mutation at position 86 in the amino acid sequences for *C. jejuni* strains. The quinolone resistance mutation at amino acid position 86, being prevalent in the clinic, modulates DNA supercoiling homeostasis in *Campylobacter*. These unique DNA sequences in the QRDR region may be useful during the investigation of an epidemiological outbreak.

An alarmingly increasing rate of resistance to quinolone resistance was reported in a neighboring country, Turkey, registering up to 74.3% (2013) resistant isolates [7,28].

The detection of mutations in the rRNA23S gene at position 2074 and 2075 associated with macrolide resistance (for erythromycin resistance) was determined in 12 strains (18%) of the 66 *C. jejuni* isolates. The data obtained by sequencing were compared and analyzed with the sequence from the *C. jejuni* NCTC 11168 23Sribosomal database, which confirmed the presence of a point mutation at position 2075 (A–G) in all 12 isolates of *C. jejuni* with high erythromycin resistance. Five isolates showed a double mutation in both position 2074 (A–C) and position 2075. Five strains showed a mutation in position 2074, and seven strains did not show this mutation.

The results are consistent with similar results due to the presence of the mutation in position 2075 for *C. jejuni* strains with increased resistance. Therefore, the validation of these tests for the detection of point mutations in *the rRNA 23 S* gene associated with erythromycin resistance was confirmed.

Results of tetracycline susceptibility testing determined that 55% of *C. jejuni* clinical isolates (36 of 66 isolates) showed antibiotic resistance, whereas 45% of isolates showed sensitivity to this class of antibiotics. Due to the long widespread use of tetracyclines, a number of determinants of resistance to this class of antibiotics have been observed in a variety of bacteria. Generally, this resistance is mediated by one of four mechanisms: efflux pumps, chemical modification of tetracyclines, ribosomal protection proteins, and mutations in rRNA [7,29]. Efflux pump and ribosomal protection genes are the most important mechanisms of tetracycline resistance. The acquisition of new tetracycline resistance genes is mostly associated with mobile components such as plasmids or transposons, which are often conjugative elements [30].

An EFSA report in 2021 issued the following percentages for resistance to erythromycin and ciprofloxacin, antibiotics used for *Campylobacter jejuni* infection in human samples: Slovenia 14.2% erythromycin resistance and 86.9% ciprofloxacin resistance; Poland 5.4% vs. 92.5%; Germany 2.2–66.6%; Lithuania 1.9–92.2%; and Romania 0.1–74.7% [31].

For both species *C. jejuni* and *C. coli*, the proportion of erythromycin resistance in Portugal is much higher than the EU median for human isolates: *C. jejuni* 3.3% vs. 1.5% and *C. coli* 52.2% vs. 12.9% in 2018–2019 [32].

A study carried out over a period of 8 years in Shanghai, China, showed a high prevalence of resistance to ciprofloxacin and erythromycin among isolates of *Campylobacter jejuni* and *Campylobacter coli*, with 96% vs. 100% resistance to ciprofloxacin and 2.5%/59.23% resistance to erythromycin [33].

Campylobacter infection remains one of the major causes of gastroenteritis worldwide, with increasing prevalence and incidence in both developed and developing countries [34].

Increasing antibiotic resistance of *Campylobacter* spp. strains isolated from both humans and animals is expected to become a significant public health problem in both developed and developing countries [35]. Most *Campylobacter* infections have been found to be self-limiting. Antibiotics may be prescribed in severe cases or in immunocompromised patients [36]. Virulence and antibiotic resistance profiles such as fluoroquinolones were observed in this study, along with macrolides and tetracyclines, classes of antibiotics that are often used first-line in the treatment of infections with *C. jejuni* strains.

An ECDC surveillance reported that the highest level of resistant *C. jejuni* isolated from broilers was detected in Romania and Cyprus [37].

Also, an increased level of resistance was detected in pig isolates, but the resistance to colistin in broilers decreased [38].

Moreover, ECDC reported in disease surveillance that the European countries with the highest percentage of reported *C. jejuni* infections in 2022 are Germany followed by Spain and Czechia, with Romania being located in the 21st place. The mean rate of notification reported by the 30 EU/EEA countries was 46.83 cases per 100,000 in the population, with the highest rate in children under 5 years old. Romania was the only country where all those with reported cases were hospitalized [39]. It is therefore necessary to undertake ongoing monitoring of both the prevalence and molecular characteristics of bacterial virulence and resistance regarding effective treatment information for *Campylobacter* infections.

The diversity of housekeeping genes for the 66 isolates of *C. jejuni* was analyzed by MLST. The allelic profiles obtained generated 38 sequence types (ST) of which 3 new *Campylobacter jejuni* STs (10589, 10595, 10686) were absent in the database used for ST typing and entered in the international database under accession numbers 106266, 106267, and 106791. Schemes for the characterization and discrimination of various bacterial isolates are essential for epidemiological, genetic, and evolutionary studies.

The study provides the first information on the circulation of a polyclonal population of *Campylobacter jejuni* at the local level. The molecular typing technique using MLST discriminates between *C. jejuni* isolates effectively and generates data that can be applied to investigate the population structure and its evolutionary mechanisms.

Schemes for the characterization and discrimination of diverse bacterial isolates are essential for epidemiological, genetic, and evolutionary studies. Ideally, these schemes generate data that are relevant to all these domains, but before the advent of high-throughput nucleotide sequencing technology, this goal proved elusive [39].

The need for appropriate molecular typing schemes for *C. jejuni* isolates is essential because this human pathogen possesses extensive reservoirs from animals and the environment, and the relationships between diseases associated with the animal population or the environment remain completely unclear [40].

This study demonstrates that the molecular typing technique by using MLST discriminates between *C. jejuni* isolates effectively and generates data that can be applied to investigate the population structure and its evolutionary mechanisms.

## Figures and Tables

**Table 1:** List of primers and reaction conditions used in this study.

Target Gene	Primer Sequence 5′-3′	Amplicon Length (bp)	Amplification Program	Reference
*CadF*	F2B:5′TGGAGGGTAATTTAGATATG3′ R1B: 5′CTAATACCTAAAGTTGAAAC3′	400	94 °C 1 min 45 °C 1 min × 30 72 °C 3 min	Konkel et al., 1999 [11]
*CeuE*	JEJ1:5′CCTGCTCGGTGAAAGTTTTG3′ JEJ2:5′GATCTTTTTGTTTTGTGCTGC3′	794	−93 °C 3 min −95 °C 30 s −57 °C 30 s × 30 −72 °C 1 min	Gonzalez et al., 1997 [12]
*virB11*	VirBF:5′GAACAGGAAGTGGAAAAACTAGC-3′ VirBR:5′TTCCGCATTGGGCTATATG-3′	708	−95 °C 2min −95 °C 30 s −52 °C 30 s × 35 −72 °C 1min	Bacon et al., 2000 [13]
*flaA*	flaAF:5′GGATTTCGTATTAACACAAATGGTGC3′ flaAR:5′CTGTAGTAATCTTAAAACATTTTG3′	1728	−94 °C 1min −45°C 1 min × 30 −72 °C 3 min	Nachamkim et al., 1993a [14]
*cdtA*	GNW:5′GGAAATTGGATTTGGGGCTATACT-3′ IVH:5′ATCACAAGGATAATGGACAAT-3′	165	94 °C 1min −42 °C 1 min × 30 −72 °C 3 min	Pickett et al., 1996 [15]
*cdtB*	VAT2: 5′GTTAAAATCCCCTGCTATCAACCA 3′ WMI-R:5′GTTGGCACTTGGAATTTGCAAGGC3′	495	−94 °C 1min −42 °C 1 min × 30 −72 °C 3 min	Pickett et al., 1996 [15]
*cdtC*	WMI-F:5′TGGATGATAGCAGGGGATTTTAAC3′ LPF-X:5′TTGCACATAACCAAAAGGAAG-3′	555	−94 °C 1min −42 °C 1 min × 30 −72 °C 3 min	Pickett et al., 1996 [15]
*GZgyrA5* *GZgyrA6*	F: ATTTTTAGCAAAGATTCTGAT R: CCATAAATTATTCCACCTGT	673	−94 °C 3 min −94 °C 1 min −50 °C 1 min × 30 −72 °C 3 min −72 °C 5 min	Gerald Zirnstei n et al.,1999. [16]
used for sequencing *GZgyrA7* *GZgyrA8*	F:TTATTATAGGTCGTGCTTTG R:TAGAAGGTAAAACATCAGGTT	673	−96 °C 2 min −96 °C 20 s −50 °C 20 s × 40 −60 °C 4 min	Gerald Zirnstei n et al., 1999. [16]
*23S RNA-F* *ERY2074-R*	F: TTAGCTAATGTTGCCCGTACCG R: AGTAAAGGTCCACGGGGTCTGG	485	−94 °C 5 min −94 °C 30 s −59 °C 30 s × 30 −72 °C 45 s −72 °C 5 min	Alonso et al., 2005 [17]
*23S RNA-F* *ERY2075-R*	F: TTAGCTAATGTTGCCCGTACCG R: TAGTAAAGGTCCACGGGGTCGC	485	−94 °C 5 min −94 °C 30 s −59 °C 30 s × 30 −72 °C 45 s −72 °C 5min	Alonso et al., 2005 [17]
used for sequencing *23S RNA-F* *23S RNA-R*	F: TTAGCTAATGTTGCCCGTACCG R: AGCCAACCTTTGTAAGCCTCCG	697	−96 °C 2 min −96 °C 20 s −50 °C 20 s × 40 −60 °C 4 min	Alonso et al., 2005 [17]
*tetO*	DMT1 DMT2	559	−95 °C 5 min −94 °C 1 min −57 °C 1 min × 30 −72 °C 1 min −72 °C 5 min	Gibreel A et al, 2004 [18]

**Table 2:** Sequence of primers for amplification and sequencing of *Campylobacter jejuni* strains.

Target Locus	Primer Amplification	Primer Sequencing	Amplicon Size
*aspA*	A9 AGTACTAATGATGCTTATCC	S3 CCAACTGCAAGATGCTGTACC	899 bp
	A10 ATTTCATCAATTTGTTCTTTGC	S6 TTCATTTGCGGTAATACCATC	
*glnA*	A1 TAGGAACTTGGCATCATATTACC	S3 CATGCAATCAATGAAGAAAC	1262 bp
	A2 TTGGACGAGCTTCTACTGGC	S6 TTCCATAAGCTCATATGAAC	
*gltA*	A1 GGGCTTGACTTCTACAGCTACTTG	S3 CTTATATTGATGGAGAAAATGG	1012 bp
	A2 CCAAATAAAGTTGTCTTGGACGG	S6 CCAAAGCGCACCAATACCTG	
*glyA*	A1 GAGTTAGAGCGTCAATGTGAAGG	S3 AGCTAATCAAGGTGTTTATGCGG	816 bp
	A2 AAACCTCTGGCAGTAAGGGC	S4 AGGTGATTATCCGTTCCATCGC	
*tkt*	A3 GCAAACTCAGGACACCCAGG	S5 GCTTAGCAGATATTTTAAGTG	1102 bp
	A6 AAAGCATTGTTAATGGCTGC	S6 AAGCCTGCTTGTTCTTTGGC	
*pgm*	A3 TCAGGGCTTACTTCTATAGG	S5 GGTTTTAGATGTGGCTCATG	1150 bp
	A4 AGCTTAATATCTCTGGCTTC	S2 TCCAGAATAGCGAAATAAGG	

**Table 3:** Detection of virulence genes by PCR for 66 isolates of *Campylobacter jejuni* from fecal samples.

Virulence Gene	No. of Positive Strains	Prevalence (%)
*CadF*	66	100
*CeuE*	66	100
*virB11*	14	21
*flaA*	66	100
*cdtA*	62	93
*cdtB*	65	98
*cdtC*	65	98

**Table 4:** Genetic diversity of MLST loci for *C. jejuni* strains.

Locus	Fragment Size (bp)	Total Number of Alleles	Allele Frequency
*aspA*	477	12	2, 7, 8, 9
*glnA*	477	14	2, 17
*gltA*	402	11	2, 5
*glyA*	507	17	2, 10 and 838 new allele
*pgm*	498	16	10, 11
*tkt*	459	16	1, 3, 5
*uncA*	489	13	5, 6 and 632 new allele

**Table 5:** Frequent *C. jejuni* isolates, identified with MLST technique from 66 analyzed strains.

*C. jejuni* Strains	Complex Type	Number	Frequency (%)
ST824	CC 257	9	13.63
ST50	CC21	5	7.57
ST51	CC443	4	6.06
ST400/ST353	CC353	4	6.06
ST2079	CC52	3	4.54
ST2066	CC48	3	4.54

## Data Availability

The raw data supporting the conclusions of this article will be made available by the authors on request.

## Supplementary Materials

The following supporting information can be downloaded at: https://www.mdpi.com/article/10.3390/pathogens13090716/s1, Figure S1: Alignment of the amino acid sequences of the QRDR region for the *gyrA* gene of *C. jejuni* isolates. The position of the Thr-86-Ile mutation was compared and analyzed with the sequence in the database accession number L04566 for *C. jejuni*; Figure S2: Alignment of the nucleotide sequences of the QRDR region for the *gyrA* gene. The presence of mutation in position 257 of the ACA to ATA codon and silent mutations in position 243, associated with fluroquinolone resistance of *C. jejuni* isolates; Figure S3: The presence of mutations in position 2074 and 2075 in *C. jejuni* strains with increased resistance to erythromycin:-the presence of the double mutation both in position 2074 (A–C) and in position 2075 (A–G); - presence of mutation in position 2075 (A–G); Figure S4: ST type and clonal complex identified from the MLST database; Table S1: *Campylobacter jejuni* strains analyzed by the MLST (*multilocus sequence typing*).
