# Supplementary material for: Genetic Diversity, Virulence, and Antibiotic Resistance Determinants of Campylobacter jejuni Isolates in Romania
Source: Pathogens. 2024 Aug 23;13(9):716. doi: 10.3390/pathogens13090716 (PMC11434920; doi:10.3390/pathogens13090716)
Supplement: Supplementary file 1 [file pathogens-13-00716-s001.zip › pathogens-3134844-supplementary.pdf]

Fig.1 Alignment of the amino acid sequences of the QRDR region for the *gyr*A gene of *C.jejuni* isolates. The position of the Thr-86-Ile mutation was compared and analyzed with the sequence in the database accession number L04566 for *C.jejuni*.

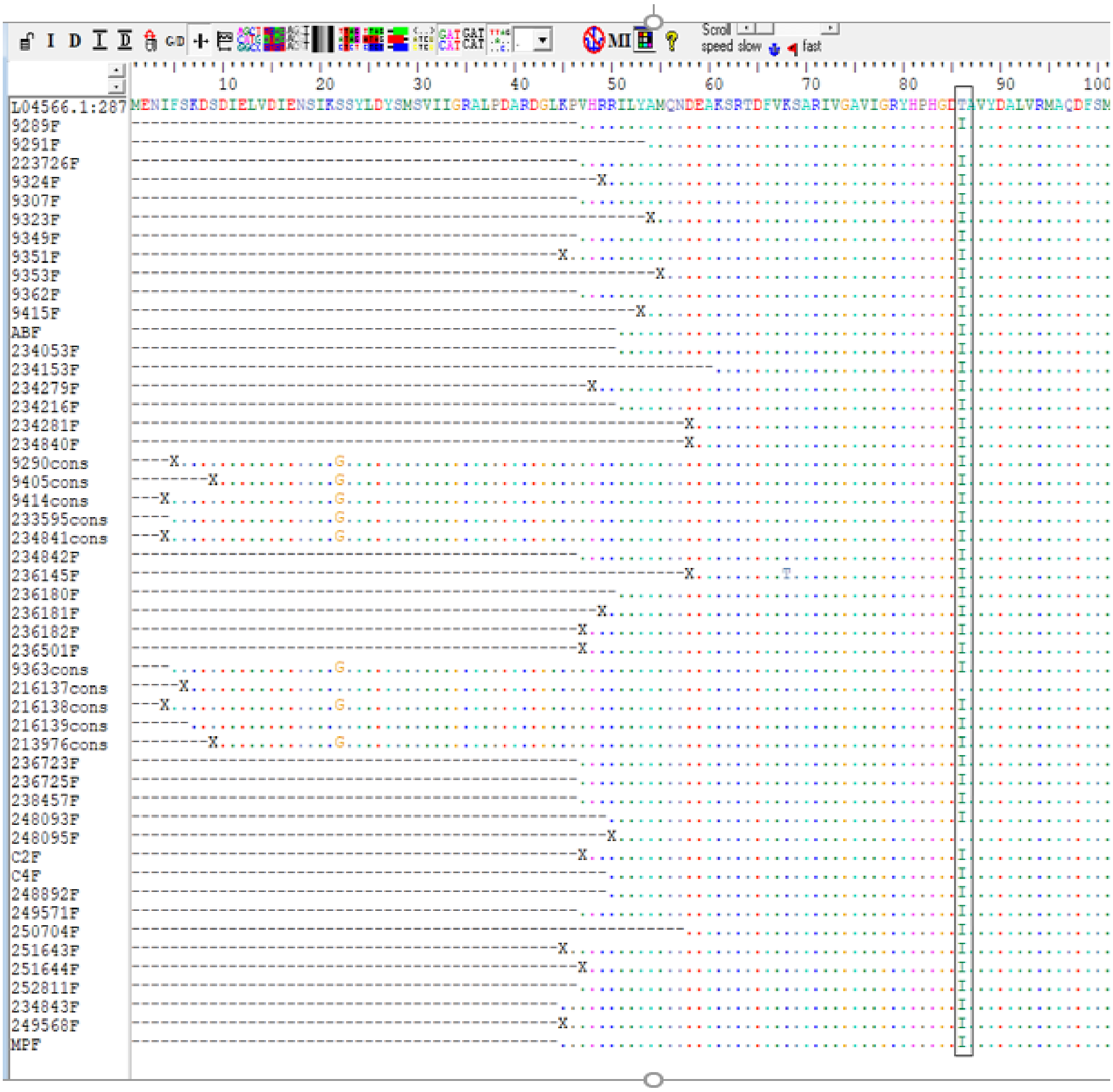

Fig.2 Alignment of the nucleotide sequences of the QRDR region for the *gyr*A gene. The presence of mutation in position 257 of the ACA to ATA codon and silent mutations in position 243, associated with fluroquinolone resistance of *C.jejuni* isolates.

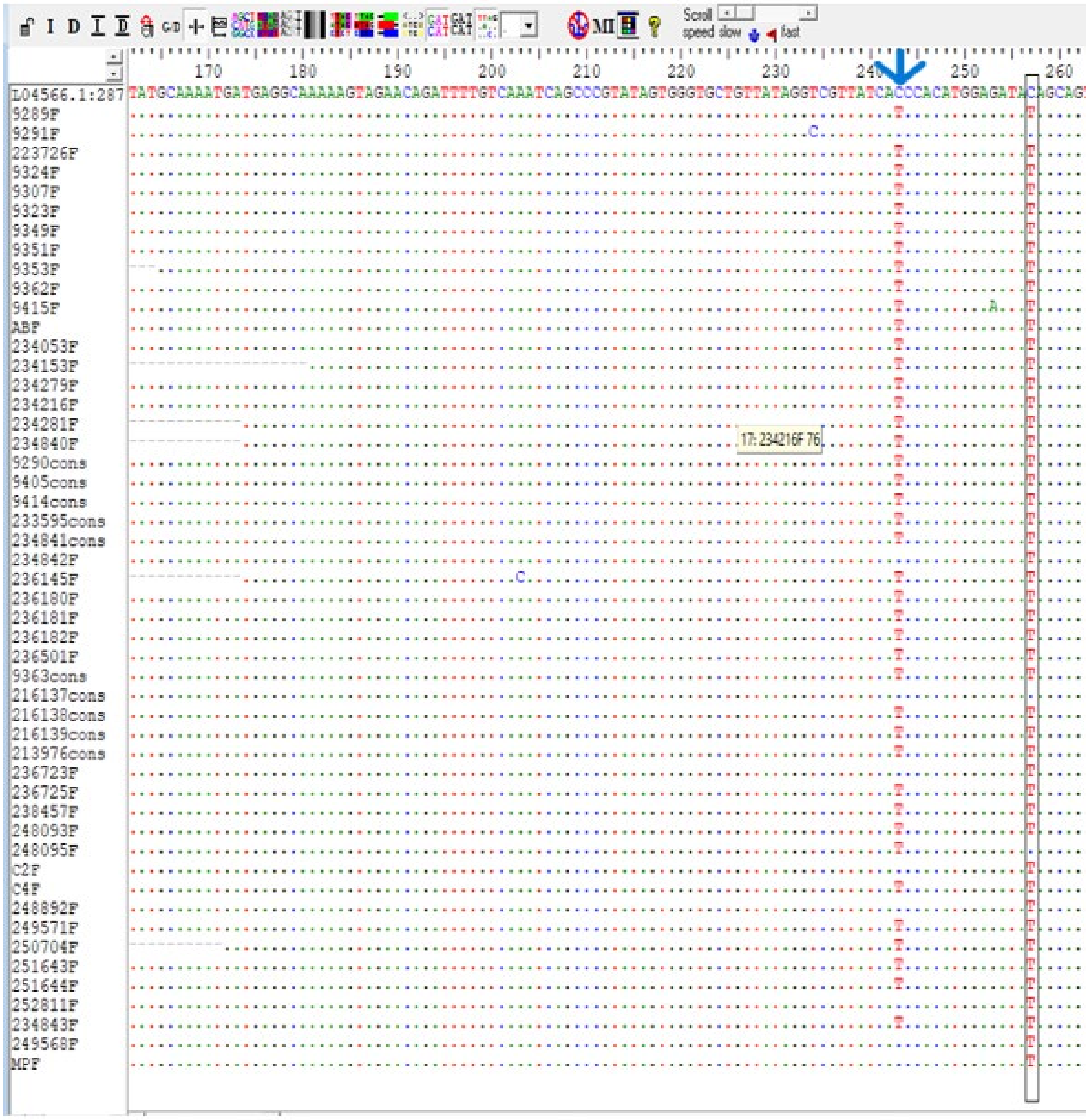

Scroll speed slow fast
170
180
190
200
210
220
230
250
260
L04566.1:287
9289F
9291F
223726F
9324F
9307F
9323F
9349F
9351F
9353F
9362F
9415F
ABF
234053F
234153F
234279F
234216F
234281F
234840F
17: 234216F 76
9290cons
9405cons
9414cons
233595cons
234841cons
234842F
236145F
236180F
236181F
236182F
236501F
9363cons
216137cons
216138cons
216139cons
213976cons
236723F
236725F
238457F
248093F
248095F
C2F
C4F
248892F
249571F
250704F
251643F
251644F
252811F
234843F
249568F
MPF

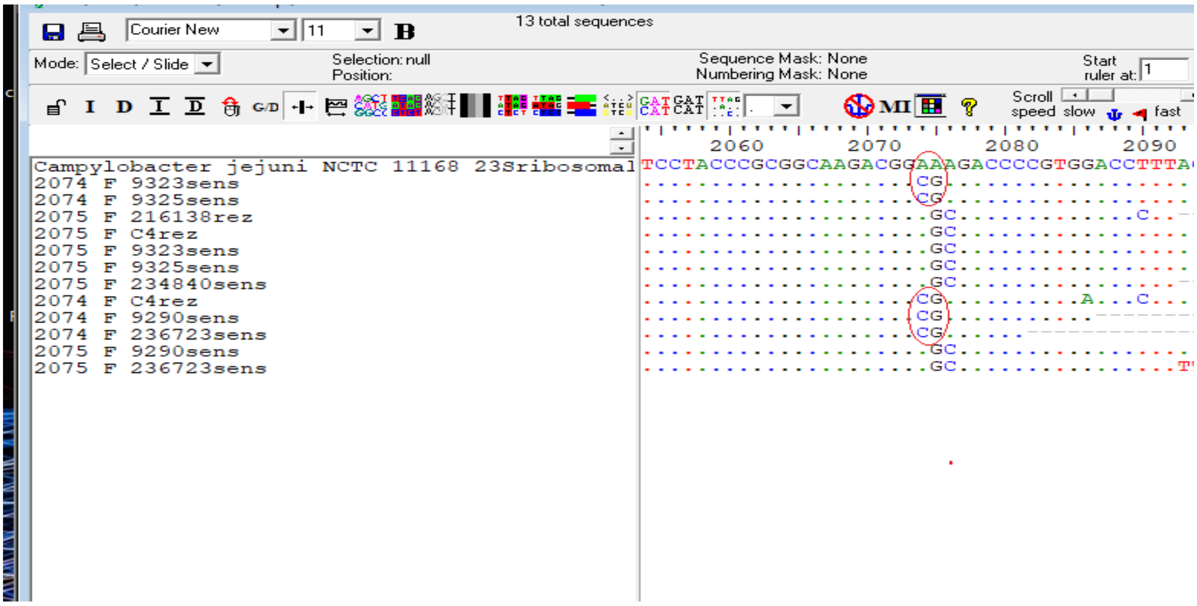

Fig.3 The presence of mutations in position 2074 and 2075 in *C.jejuni* strains with increased resistance to erythromycin:-the presence of the double mutation both in position 2074 (A-C) and in position 2075 (A-G); - presence of mutation in position 2075 (A-G).

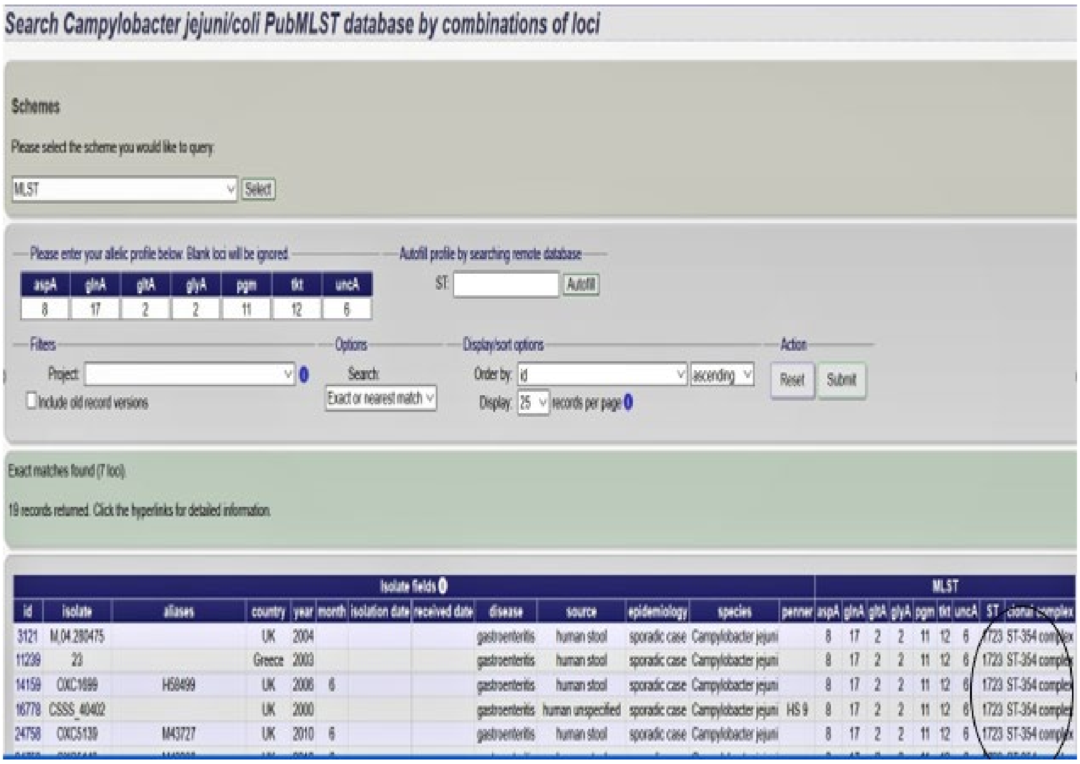

Fig. 4. ST type and clonal complex identified from the MLST database.

Table S1. *Campylobacter jejuni* strains analyzed by the MLST (*multilocus sequence typing*).

Nr.crt.	ID tulpina *C.jejuni*	aspA/alela	gln/alela	gltA/alela	glyA/alela	pgm/alela	tkt/alela	uncA/alela	ST	CC
1	213976	8	17	2	2	11	12	6	1723	ST-354 complex
2	214590	14	17	5	2	11	3	6	356	ST-353 complex
3	216137	1	3	6	6	3	3	3	22	ST-22 complex
4	216138	9	2	2	2	11	5	6	824	ST-257 complex
5	216139	8	17	5	2	10	59	6	400	ST- 353 complex
6	9289	1	2	42	450	10	9	59	5590	-
7	9290	73	21	2	10	86	3	6	6	ST-52 complex
8	9291	4	7	10	4	125	7	1	3940	ST-45 complex
9	223726	2	1	12	3	2	1	5	50	ST-21 complex
10	9307	8	17	5	2	10	59	6	400	ST-353 complex
11	9308	2	1	12	3	2	1	5	50	ST-21 complex
12	9323	2	17	52	10	11	3	6	6813	-
13	9324	9	2	2	2	11	5	6	824	ST-257 complex
14	9325	14	4	1	4	19	1	5	2079	ST-48 complex
15	9349	14	4	1	4	19	1	5	2079	ST-48 complex
16	9350	14	4	1	4	19	1	5	2079	ST-48 complex
17	9351	8	10	10	2	11	12	6	2992	ST-354 complex
18	9353	9	2	2	2	11	5	6	824	ST-257 complex
19	9362	9	2	2	2	11	5	6	824	ST-257 complex
20	9363	8	2	4	62	4	5	6	2254	ST-257 complex
21	Margutoiu Patrick	2	1	12	3	2	27	5	50	ST-21 complex
22	9404	9	2	2	2	11	5	6	824	ST-257 complex
23	9405	7	17	5	2	10	3	6	353	ST-353 complex
24	9414	8	10	2	2	11	12	6	354	ST-354 complex
25	9415	2	17	52	10	11	3	6	6813	-
26	Andreea Benga	7	78	42	95	14	12	8	534	-
27	233594	9	25	2	10	22	3	6	52	ST-52 complex
28	233595	8	17	5	2	10	59	6	400	ST-353 complex
29	234053	7	17	5	2	10	3	6	353	ST-353 complex
30	234153	7	17	2	15	23	3	12	51	ST-443 complex
31	234279	9	10	5	10	22	3	6	2066	ST-52 complex
32	234213	7	84	5	10	119	178	26	1911	-
33	234215	8	7	5	4	307	7	6	1003	ST-45 complex
34	234216	8	2	4	62	4	5	6	2254	ST-257 complex
35	234281	24	2	4	838*	10	491	1	10589*	ST-464 complex
36	234840	2	1	12	3	2	1	5	50	ST-21 complex
37	234841	7	2	5	2	10	3	6	353	ST-353 complex
38	234842	2	1	12	3	2	1	5	43	ST-21 complex
39	234843	24	2	5	53	23	491	1	904	ST-607 complex
40	234738	316	17	2	15	23	491	12	51	ST-443 complex
41	235627	9	25	2	10	22	706	632*	10595*	ST-52 complex
42	236145	8	17	5	2	10	59	6	400	ST-353 complex
43	236179	8	2	5	53	11	3	1	607	ST-607 complex
44	236180	7	53	2	10	11	3	3	574	ST-574 complex
45	236181	2	17	52	10	11	3	6	6813	-
46	236182	9	2	2	2	11	5	6	824	ST-257 complex
47	236501	9	264	2	312	11	5	6	824	ST-257 complex
48	236722	2	21	10	37	2	1	5	206	ST-206 complex
49	236723	2	1	1	3	2	226	5	1943	ST-21 complex
50	236725	7	17	2	15	23	3	12	51	ST-443 complex
51	238457	7	17	2	15	23	3	12	51	ST-443 complex
52	248093	9	2	2	2	11	5	6	824	ST-257 complex
53	248095	9	2	2	2	11	5	6	824	ST-257 complex
54	C2	24	115	4	28	278	29	23	5397	-
55	C3	2	4	1	93	258	491	6	3076	ST-658 complex
56	C4	8	2	2	212	11	253	147	7536	-
57	248786	7	17	2	15	23	3	12	51	ST-443 complex
58	248891	2	17	2	3	2	1	5	883	ST-21 complex
59	248892	2	1	12	3	2	1	5	50	ST-21 complex
60	249568	10	27	59	19	10	5	7	1775	ST-403 complex
61	249571	346	2	3	4	5	9	3	6532	ST-42 complex
62	250704	8	17	5	2	10	59	6	400	ST-353 complex
63	251643	9	10	5	10	22	3	6	2066	ST-52 complex
64	251644	9	10	5	10	22	3	6	2066	ST-52 complex
65	252811	2	2	5	2	340	1	57	10686*	-
66	252813	9	17	5	2	10	59	6	6461	ST-353 complex
	* new allele and ST									
